# Role of time perspectives and self-control on well-being and ill-being during the COVID-19 pandemic: A multiple mediation model

**DOI:** 10.1186/s40359-022-00933-2

**Published:** 2022-10-27

**Authors:** Min Zong, Dan Dong, Zhizun Yang, Yi Feng, Zhihong Qiao

**Affiliations:** 1 Faculty of Psychology, Beijing Normal University, Beijing, China; 2 Mental Health Center, China Foreign Affairs University, Beijing, China; 3 Mental Health Center, Central University of Finance and Economics, No. 39 South College Road, Haidian District, 100081 Beijing, China

**Keywords:** Time perspective, Self-control, Well-being, Ill-being, COVID-19

## Abstract

**Background:**

A growing body of evidence indicates that the outbreak of COVID-19 has had a significant influence on individuals’ cognition, emotion, and psychological health. This study aims to explore the effect of the association between time perspectives and self-control on the well-being and ill-being among college students in China during the COVID-19 pandemic.

**Methods:**

We conducted an online survey involving 1,924 participants in mainland China during the outbreak of COVID-19. A series of self-rating questionnaires measuring the perceived impact of COVID-19, time perspectives, self-control, as well as the statuses of well-being and ill-being were administered. Multiple indirect effects of time perspectives and self-control on well-being and ill-being were analysed through structural equation modelling.

**Results:**

The present-hedonistic time perspective (an orientation on immediate impulses of pleasure) meditated the effects of perceived impacts on both well-being and ill-being, and the future time perspective (considering the outcomes of actions and decisions) mediated the effects on well-being. Moreover, the mediating effects were further mediated by self-control. Specifically, the impact of the future time perspective on ill-being was fully mediated by self-control (*β* = 0.01, p < 0.01).

**Conclusion:**

Based on the results, it is evident that the present-hedonistic time perspective, the future time perspective, and self-control are related to higher levels of well-being and lower levels of ill-being, thereby providing further insight into the theoretical framework of time perspectives during the COVID-19 pandemic. Additionally, our findings provide practical implications for psychological interventions during the on-going COVID-19 pandemic, focusing on the effects of time perspectives and self-control on the well-being and ill-being of different individuals.

**Supplementary Information:**

The online version contains supplementary material available at 10.1186/s40359-022-00933-2.

## Background

The COVID-19, which occurred worldwide throughout 2020, has become a public health concern worldwide and remains a global pandemic [1, 2]. A growing body of evidence indicates that the outbreak of COVID-19 significant influence on individuals’ cognition, emotion, and psychological health [2–5]. Moreover, the outbreak of COVID-19 may dramatically alter how we perceive time and view our futures [6].

Time perspective (TP), which is a significant concept in social psychology, refers to the individual unconscious view and cognitive process into past, present, and future temporal frames [7], which is of significant importance to well-being and distress [8]. Although there is a growing interest in TPs, the affecting factors [9, 10] and effects of TPs on well-being remain unclear [8, 11]. The COVID-19 pandemic presents an opportunity to explore the potential mediating role of TPs in mental health. This study aims to investigate the impact of TPs on mental health during the COVID-19 pandemic by considering well-being and ill-being simultaneously. In theory, this study enriches the theoretical framework of TPs, especially the changes in TPs and the mediating role of TPs on mental health during the COVID-19 pandemic. In practice, this study also provides further insight into clinical practice and public health management during the pandemic from the perspective of TPs. In addition, exploring the protective time perspective for well-being also provides some reference for other countries to prepare for the next pandemic.

### Perceived impact of COVID-19 on mental health

Based on the stress coping theory [12, 13], the perceived threat or uncertainty of external events, such as the COVID-19 pandemic, could result in maladaptive cognitions, which initiate coping responses and result in negative affectivity and mental health consequences [4, 14, 15]. However, the dual-factor model of mental health considers that mental health is constructed by two separate factors (i.e., well-being and ill-being), rather than a one-dimensional structure. In other words, well-being (i.e., satisfaction with one’s life) [16, 17] and ill-being (i.e., negative psychological maladjustment) [18–20] together construct the full picture of mental health [21]. Several studies have explored the effects and mechanisms of the pandemic on well-being [2, 15, 22] and psychological distress [3, 4], but to the best of our knowledge, there are few studies exploring well-being and ill-being simultaneously. This study attempts to combine well-being and ill-being as mental health outcomes, and it investigates the role of the perceived impact of COVID-19 on mental health and explores its underlying mechanism through the mediating roles of TPs and self-control.

### Time perspectives and mental health during the COVID-19 pandemic

An individual’s TP comprises their views on the past, present, and future. It represents a cognitive predilection towards a specific temporal condition [23], including changes in the environment, stress, and culture [24], and it has a prominent influence on human ideology, feelings, and behaviours [11]. Individual’s TPs are divided into five categories according to Zimbardo’s time perspective theory [23]: past-positive (looking fondly on the past), past-negative (involves negative views on the past), present-fatalistic (involves the belief that life is out of one’s control), present-hedonistic (an orientation on immediate impulses of pleasure), and future (considers the outcomes of actions and decisions) TPs. Different TPs have unique contributions to well-being and mental health [8].

The past-positive, past-negative, and present-fatalistic TPs generally measure attributes related with the long-term life changes, such as trauma [25, 26] and nostalgia[10]. According to the Life History Theory, the perceived uncertainty of external environmental could result in their choice of long-term or short-term survival strategies, leading to different mental health outcomes [10, 27]. PHTP and FTP were psychologically represented the essence of life history trade-offs [10], presenting the motivational process of short-term survival strategy (such as pleasure seeking) and long-term survival strategy (such as the future goals pursuing) [10, 23]. Previous studies have shown that PHTP and FTP partly mediate the relationship between perceptions of local social conditions and risky behaviours [10]. The COVID-19 has brought great uncertainty to human lives and may affect functional development adaptation as indicated by time perspective. Ogden demonstrated that people’s experiences of time were significantly changed by the social and physical distancing measures enforced during the COVID-19 lockdown in the UK [5]. Moreover, PHTP and FTP showed different effects on mental health[9, 28, 29]. Individuals with the PHTP tend to experience a more positive affect [23, 30]. However, this could diminish their well-being by increasing risk-taking and aggressive behaviors [31]. Although the FTP could be used as a predictor of a higher level of life satisfaction and increased subjective happiness [32], pursuing future goals may decrease enjoyment among individuals with such a TP [33].Therefore, in the context of social isolation and great uncertainty caused by COVID-19, it may be difficult for people to maintain an FTP in the pandemic, rather, they are more likely to choose PHTP, thereby affecting their well-being and ill-being. This study focuses on the present-hedonistic TP (PHTP) and the future TP (FTP). We assume that the PHTP and the FTP may affect the relationships between the perceived impact of COVID-19 and mental health.

### Relationship between time perspectives, self-control, and mental health

The dual-pathway framework theory suggests that TPs might affect the well-being both directly (the top-down path) and indirectly (the bottom-up path) [8]. Therefore, other mediating variables should also be considered in this study, such as self-control.

Self-control is considered an ability or a self-regulatory process that overrides undesired but cheerful impulses/actions to advance the realisation of distal goals [34], thereby contributing to the promotion of well-being [35, 36]. Self-control is highly associated with the PHTP and the FTP [23, 37]. Individuals with the PHTP have low levels of self-control, whereas those with the FTP have increased levels of perceived self-control [28, 29, 37]. Because of the close association between the PHTP, the FTP, and self-control, several studies have indicated that self-control mediates the effect of PHTP or FTP on mental health problems, including procrastination and internet addiction [29], and physical health (e.g. BMI) [38]. However, it remains unclear whether self-control served as a mediator between TPs and mental health during the outbreak of COVID-19.

### Aims of this study

**Fig. 1:**
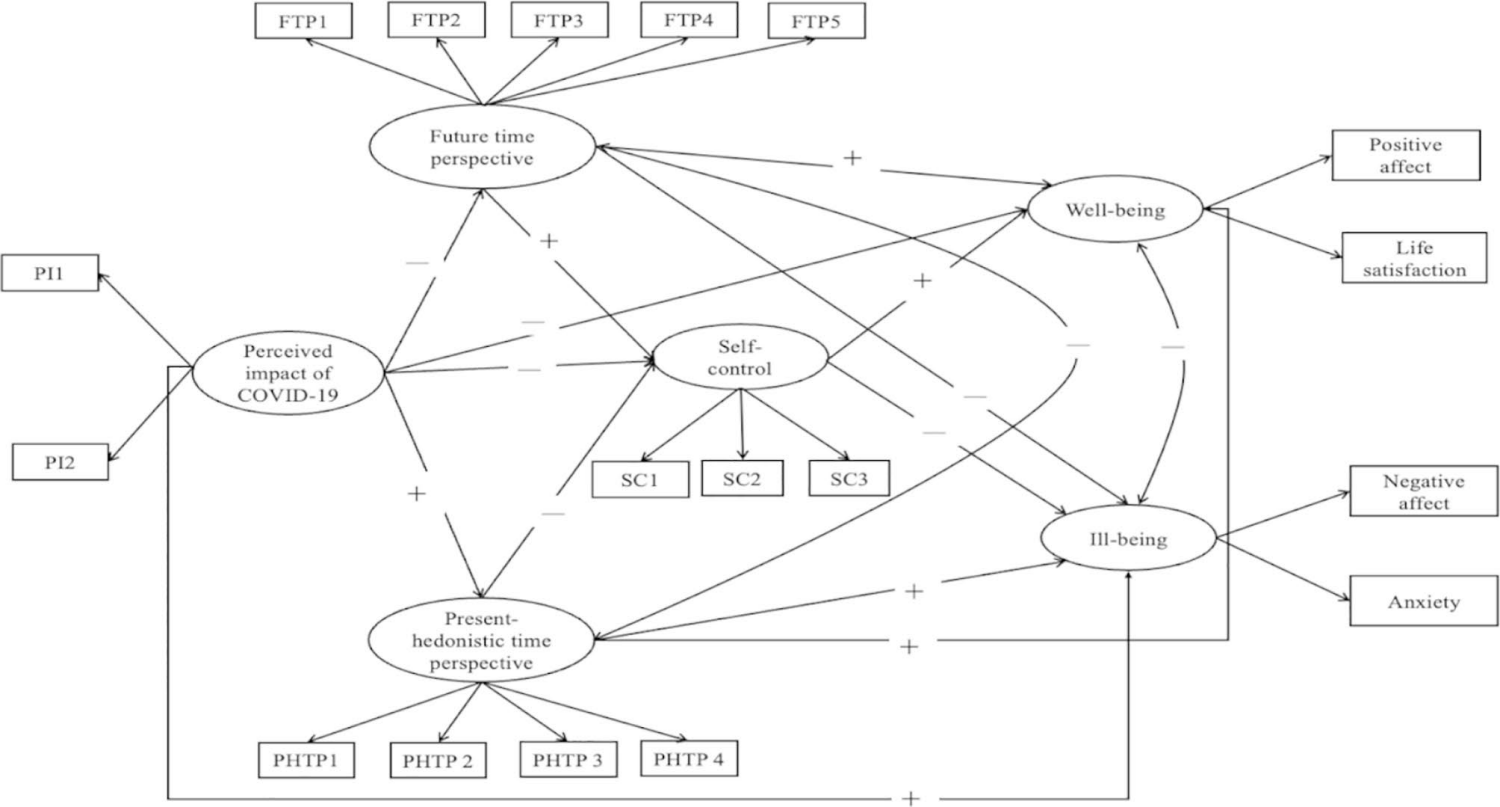
The hypothesised model *Note*. The plus and minus signs present the positive and negative predictive effects of the pathways, respectively.

This study aims to formulate a theoretical model of the influence of TPs (e.g. PHTP and FTP) on mental health during the COVID-19 pandemic and to clarify the mechanism through a structural equation model (SEM). Essentially, we hypothesised that the perceived impact of COVID-19 negatively predicts well-being and positively predicts ill-being through multiple mediations of the FTP, the PHTP, and self-control (see Fig. 1).

## Methods

### Participants and sampling

This study was conducted from 30 April to 11 May, 2020, after the peak stage of the COVID-19 pandemic in China. At that time, the number of new local infections was 0–12 per day, but college students remained taking online courses at home. Data was collected from Chinese university students through the online survey platform ‘Wenjuanxing’ using simple cluster sampling via online questionnaires. A variety of electronic devices, such as laptops and smartphones, allowed for students to complete the questionnaires.

Participants were required to complete all items before submitting, and to avoid data duplication, only one questionnaire could be completed from each IP address. All questionnaires were distributed by university teachers from 20 different universities, and compulsory questions involving university and major names were included in the questionnaires to ensure that all the participants were university students. One attention check question was added to filter valid questionnaires because incomplete answers were not allowed by the questionnaire program. Before the investigation, participants were notified of the aims and process of this study through an online notification. Each participant provided electronic written informed consent before participating in the survey. Participants that did not meet the following criteria were excluded: (1) not Chinese students or not staying in mainland China during the COVID-19 pandemic, (2) students who were or had family members infected with COVID-19, and (3) students who failed the attention check question (e.g. ‘Please choose the B option directly in this item’). In structural equation models, the minimum sample size is 10 times the number of estimated parameters [39]. A total of 70 estimated parameters were included in this study; thus, the minimum sample size required for this study is 700. A total of 2,246 students participated in this survey, 1,924 participants met the criteria, with a completion rate of 85.66%.

### Measures of variables

#### Perceived impact of COVID-19

Participants were required to rate the extent to which their lives had been impacted by COVID-19 through six items, namely, study, application for employment/internship, examination, love, friendship, and entertainment and leisure (i.e., How much have your studies been impacted by COVID-19), using a 5-point Likert scale (1 = *large positive impac*t; 5 = *large negative impact*). Because it was a self-developed scale, exploratory factor analysis (EFA) was used and two factors (learning and leisure) were extracted. A two-factor confirmatory factor analysis (CFA) model indicated a good model fit. Detailed EFA and CFA results were provided in the supplementary material. The Cronbach’s α of the two factors (i.e., the academic factor and the life factor) were 0.82 and 0.72.

#### Present-hedonistic and future time perspectives

Self-reported PHTP and FTP were evaluated using the Zimbardo Time Perspective Inventory (ZTPI) [23]. The Chinese version was validated, with Cronbach’s *α* = 0.57 ~ 0.76 [40], for 25 items. PHTP was measured using 4 items, for example, ‘I do things impulsively’. FTP was measured using 5 items, for example, ‘I complete projects on time by making steady progress’. Participants were asked to respond on a 5-point Likert scale (1 = ‘very untrue of me’, 5 = ‘very true of me’). In our study, Cronbach’s *α* was 0.81 for PHTP and 0.83 for FTP.

#### Self-control

A revised Chinese version of the Self-Control Scale [41] with 19 items [42] was used to assess self-control in this study. Responses were rated on a 5-point Likert scale ranging from ‘1 = *not at all*’ to ‘5 = *very much so*’. For example, “People can count on me to stay on schedule”. It is comprised of 5 factors (i.e., self-discipline, deliberate non-impulsive action, healthy habits, self-control in the context of work ethic, and reliability) [43]. The Cronbach’s *α* for each factor in this study were 0.59 ~ 0.82.

#### Well-being

Well-being, especially hedonic happiness, includes pleasure, happiness, satisfaction with life [44], and the absence of negative affect [45]. In this study, we constructed the latent variable ‘well-being’ as an outcome variable based on life satisfaction and positive affect [46] considering that the absence of negative affect does not ensure that there are psychological assets [47].

The Satisfaction with Life Scale used in this study was compiled by Diener et al., [48]. Participants’ life satisfaction was assessed using the Chinese version [49]. It includes 5 items (i.e., ‘in the majority of ways my life is close to my ideal’) rated on a 7-point Likert scale (from 1 = ‘*strongly disagree*’ to 7 = ‘*strongly agree*’). Cronbach’s α was 0.82 in this study.

Positive affect was measured through the Positive and Negative Affect Scale (PANAS). The Positive Affect (PA) scale, which comprises 10 positive affect items (i.e., enthusiastic and inspired), was used in this study. Participants were required to rate each item on a 5-point Likert scale (from 1 = ‘*very slightly to not at all*’ to 5 = ‘*extremely*’) to rank the extent to which they felt the positive emotion over the previous two weeks. In this study, the Cronbach’s *α* was 0.92, with higher scores showing higher levels of positive emotions.

#### Ill-being

Ill-being comprises negative psychological conditions or characteristics. The latent variable ‘ill-being’ was constructed on the basis of anxiety and negative affect in this study [50].

Anxiety symptoms [51] were assessed using the Generalised Anxiety Disorder Scale (GAD-7). The Chinese version of the GAD-7 demonstrated good reliability (Cronbach’s *α* = 0.89) [52]. This version of the GAD-7 includes 7 items (i.e., ‘Feeling nervous and anxious’) that require the respondents to rate the frequency with which they experienced anxiety over the previous two weeks on a 4-point Likert scale (1 = ‘*not at al*l’, 2 = ‘*some days*’, 3 = ‘*more than half the days*’, and 4 = *‘almost every day*’). In this study, the Cronbach’s *α* was 0.94, with higher grades indicating severe anxiety symptoms.

Negative Affect was also measured using the PANAS. The Negative Affect (NA) scale, which comprises 10 negative affect items (i.e., afraid and distressed), was derived from the PANAS [53]. In this study, Cronbach’s *α* was 0.92, with higher grades showing a significant negative affect.

#### Demographics

Sociodemographic characteristics were collected, including age, major, grade and place of birth. All categorical variables were coded as dummy variables. Sex was coded as 0 = male, 1 = female. Dummy variable major 1 was coded as 0 = literature, 1 = economics, major 2 was coded as 0 = literature, 1 = science, major 3 was coded as 0 = literature, 1 = engineering, major 4 was coded as 0 = literature, 1 = art, major 5 was coded as 0 = literature, 1 = physical education, major 6 was coded as 0 = literature, 1 = others. Dummy variable grade 1 was coded as 0 = freshmen, 1 = sophomore, grade 2 was coded as 0 = freshmen, 1 = junior, grade 3 was coded as 0 = freshmen, 1 = Senior and above. Dummy variable place of birth 1 was coded as 0 = city, 1 = Town, place of birth 2 was coded as 0 = city, 1 = Country.

### Data analysis

SPSS 24.0 and Mplus 8.0 were applied to organise and analyse the data. Descriptive statistics, Spearman correlation analyses, and SEM were used to examine the hypothesised multiple mediating effects. All variables in the SEM model were estimated as latent variables. Perceived impact of COVID-19 was estimated by the two extracted factors. Well-being was estimated by life satisfaction and positive affect, and ill-being was estimated by anxiety and negative affect. PHTP and FTP were estimated by four and five items, respectively. Due to the large item number of the self-control variable, item parcelling approach in CFA was applied to simplify the model, increase the stability of parameter estimates, and provide greater power by reducing the number of factors [54–57]. Three parcels were constructed to estimate self-control variable using the domain representative technique, in which items from each dimension were combined until no items remained [29, 56, 58] (Detailed results of item parcelling see the supplementary material). The criteria of goodness-of-fit parameters were CFI ≥ 0.90, TLI ≥ 0.90, RMSEA ≤ 0.08, and standardized root mean square residual (SRMR) ≤ 0.08 [59]. The value of significance was 0.05 in this study. The mediation effect [60] was examined through bootstrapping, with 95% confidence intervals (CIs) in our analysis.

## Results

### Demographic statistics and correlations

Of the 1,924 participants, the average age was 19.58 years (*SD* = 1.52), ranging from 17 to 38 years of age; 704 (36.60%) were males; 820 (42.62%) were born in urban areas; 1347 (70%) were freshmen; and 714(37.11%) majored in social science (see Table 1).

**Table 1:** Summary of sociodemographic characteristics (*N* = 1,924)

Variables	***n*** (%)
*Mean* Age (*SD*)	19.58 (1.52)
Sex	
Male	704 (36.6%)
Female	1220 (63.4%)
Major	
Literature, Philosophy, Law, Education, History	714 (37.1%)
Economics, Management Science	446 (23.2%)
Science	135 (7.0%)
Engineering Science	441 (22.9%)
Art	147 (7.6%)
Physical Education	19 (1.0%)
Others (e.g., Medicine, Agriculture)	22 (1.1%)
Grade	
Freshmen	1347 (70%)
Sophomore	270 (14%)
Junior	207 (10.8%)
Senior and above	100 (5.2%)
Place of birth	
City	820 (42.6%)
Town	469 (24.4%)
Country	635 (33.0%)

The results showed that there were significant associations between well-being (i.e., life satisfaction and positive affect) and the perceived impacts of COVID-19 (*r* = − 0.19; *r* = – 0.13) on FTP (*r* = 0.30; *r* = 0.37), PHTP (*r* = − 0.15; *r* = − 0.10), and self-control (*r* = 0.29; *r* = 0.23). The associations between ill-being (i.e., anxiety and negative affect) and the perceived impacts of COVID-19 (*r* = 0.20; *r* = 0.15) on FTP (*r* = − 0.18; *r* = − 0.25), PHTP (*r* = 0.38; *r* = 0.38), and self-control (*r* = − 0.40; *r* = − 0.42) were also significant. Table 2 shows the detailed associations among the main variables.

**Table 2:** Spearman correlations between the main variables (*N* = 1,924)

Variables	1	2	3	4	5	6	7	8	9	10	11	12	13	14	15	16	17
1 Sex (1)	1																
2 Age	− 0.09**	1															
3 Major (1)	0.14**	− 0.02	1														
4 Major (2)	− 0.20**	0.01	− 0.15**	1													
5 Major (3)	− 0.44**	− 0.00	− 0.30**	− 0.15**	1												
6 Major (5)	− 0.02	0.00	− 0.06*	− 0.03	− 0.05**	1											
7 PB (2)	0.01	0.07**	− 0.18**	0.05*	-0.01	0.06**	1										
8 Grade (2)	− 0.03	0.42**	0.10**	− 0.05	0.08**	− 0.03	− 0.05*	1									
9 Grade (3)	0.07**	0.39**	− 0.06**	− 0.06**	− 0.13**	− 0.02	− 0.09**	− 0.08**	1								
10 PIA	− 0.08**	0.15**	− 0.01	0.01	0.06**	0.01	− 0.01	0.17**	0.04	1							
11 PIL	− 0.13**	0.08**	− 0.04	0.02	0.05*	− 0.02	− 0.04	0.00	0.04	0.39**							
12 FTP	0.09**	0.05*	0.02	− 0.01	− 0.07**	− 0.02	− 0.05*	0.07**	0.11**	− 0.07**	− 0.07**	1					
13 PHTP	0.03	− 0.02	− 0.06**	0.04	− 0.01	− 0.01	0.10**	0.00	− 0.08**	0.14**	0.14**	− 0.23**	1				
14 SC	− 0.02	− 0.00	0.01	− 0.04	0.01	0.01	− 0.04	− 0.00	0.06**	− 0.22**	− 0.19**	0.35**	− 0.65**	1			
15 LS	0.01	− 0.01	0.03	− 0.02	− 0.06**	− 0.02	− 0.08**	− 0.02	0.06*	− 0.20**	− 0.19**	0.30**	− 0.15**	0.29**	1		
16 PA	− 0.03	− 0.02	0.00	− 0.01	− 0.02	0.02	− 0.07**	− 0.01	0.01	− 0.12**	− 0.13**	0.37**	− 0.10**	0.23**	0.45**	1	
17 Anxiety	− 0.04	0.03	0.01	0.05*	0.05*	− 0.05*	0.02	0.09**	− 0.03	0.20**	0.20**	− 0.18**	0.38**	− 0.40**	− 0.21**	− 0.20**	1
18 NA	− 0.08**	0.06**	− 0.05*	0.05*	0.06**	− 0.00	0.05*	0.05*	− 0.02	0.13**	0.15**	− 0.25**	0.38**	− 0.42**	− 0.20**	− 0.17**	0.58**
*M*		19.57								11.50	9.94	17.53	10.39	60.13	19.80	31.40	12.00
*SD*		1.58								2.44	2.06	3.14	3.04	10.73	5.26	6.58	4.67

*Note*. All categorical variables were coded as dummy variables. Only the coded categories with significant correlations were presented in the above table for the well-formatted layout. Sex was coded as 0 = male, 1 = female. Major1 was coded as 0 = literature, 1 = economics, major 2 was coded as 0 = literature, 1 = science, major 3 was coded as 0 = literature, 1 = engineering, major 5 was coded as 0 = literature, 1 = physical education, PB: Place of birth; PB 2 was coded as 0 = city, 1 = Country, grade 2 was coded as 0 = freshmen, 1 = junior, grade 3 was coded as 0 = freshmen, 1 = Senior and above. PIA: Perceived impact of COVID-19 academic; PIL: Perceived impact of COVID-19 life; FTP: Future time perspective; PHTP: present-hedonistic time perspective; SC: self-control; LS: life satisfaction; PA: positive affect; NA: negative Affect

*p < 0.05, **p < 0.01

### Mediating effects of time perspectives and self-control

We examined the multiple mediating effects of TPs (e.g., present-hedonistic TP and future TP) and self-control related to the perceived impact of COVID-19 and psychological health (e.g., well-being and ill-being) by applying the following two steps.

Firstly, the direct role of the perceived impact of COVID-19 on the latent variables of both well-being and ill-being was acceptable (*χ*^*2*^*/df* = 4.36, CFI = 0.94, TLI = 0.90, RMSEA = 0.04, SRMR = 0.04). The results showed that the perceived impact of COVID-19 had a direct negative prediction of well-being (*β* = − 0.31, *p* < 0.001) and a positive prediction of ill-being (*β* = 0.24, *p* < 0.001).

**Fig. 2:**
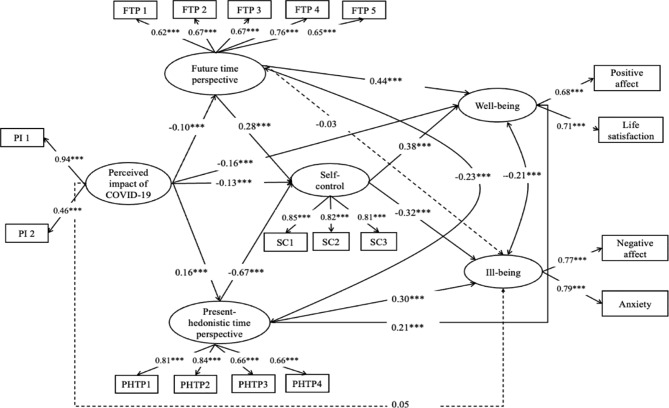
The final structural equation model *Note*. The solid lines represent significant predictive effects. The dashed line shows that the predictive effects were insignificant: ***p < 0.001.

Secondly, as shown in Fig. 2, the mediating model was examined to test the indirect influence by constructing an SEM. Sex, age, major, place of birth, and grade were stipulated as covariates in the model. The model showing the various mediating effects conformed to the data well (*χ*^*2*^*/df* = 5.28, CFI = 0.92, TLI = 0.90, RMSEA = 0.05, SRMR = 0.04).

As shown in Table 3, increased perceived negative impacts of COVID-19 predicted lower well-being via three indirect paths through the FTP (*β* = − 0.04, *p* < 0.001), PHTP (*β* = 0.04, *p* < 0.01), and self-control (*β* = − 0.05, *p* < 0.001) separately, and two multiple indirect paths through the FTP and self-control (*β* = − 0.01, *p* < 0.01) or the PHTP and self-control (*β* = − 0.04, *p* < 0.001). Additionally, a higher perceived negative impact of COVID-19 positively predicted ill-being via two indirect paths through the PHTP (*β* = 0.05, *p* < 0.001) and self-control (*β* = 0.04, *p* < 0.001) and via two multiple indirect paths through the FTP and self-control (*β* = 0.01, *p* < 0.01) or the PHTP and self-control (*β* = 0.04, *p* < 0.001). Self-control acted as a chain mediation variable, completely mediating the variation from the FTP to ill-being. From Perceived Impact of COVID-19 to ill-being is completely mediated by FTP, PHTP and self-control. The total indirect effects explained 10.9% of the variance in well-being and 13.80% of the variance in ill-being.

**Table 3:** Standardized direct, indirect, and total effects for the mediation model (*N* = 1,924)

Model pathways	Standardized ***β***	95% ***CI***		***p*** value
		*Lower* 5%	*Upper* 5%	
***Well-being***				
Direct effect	–0.31	–0.38	–0.25	0.000
Indirect effects	–0.11	–0.14	–0.07	0.000
PI→FTP→Well-being	–0.04	–0.07	–0.02	0.004
PI→PHTP→Well-being	0.04	0.01	0.06	0.008
PI→SC→Well-being	–0.05	–0.07	–0.03	0.000
PI→FTP→SC→Well-being	–0.01	–0.02	–0.00	0.007
PI→PHTP→SC→Well-being	–0.04	–0.06	–0.02	0.001
***Ill-being***				
Direct effect	0.25	0.19	0.31	0.000
Indirect effects	0.14	0.10	0.18	0.000
PI→FTP→Ill-being	0.00	0.00	0.01	0.425
PI→PHTP→Ill-being	0.05	0.02	0.07	0.000
PI→SC→Ill-being	0.04	0.03	0.06	0.000
PI→FTP→SC→Ill-being	0.01	0.00	0.02	0.010
PI→PHTP→SC→Ill-being	0.04	0.02	0.05	0.001

*Note*. PI: Perceived impact of COVID-19; FTP: Future time perspective; PHTP: Present-hedonistic time perspective; SC: Self-control

## Discussion

This study is among the few researches that examine the changes in TPs and the effects of TPs and its relationship with self-control on well-beings and ill-beings during the COVID-19 pandemic.

The results showed that a higher perceived negative impact of COVID-19 may be predicted as decreasing well-being and increasing ill-being. Notably, the increased effect of perceived impact of COVID-19 on ill-being was fully mediated by FTP, PHTP, and self-control. These results are consistent with previous studies during the outbreak stage of the COVID-19 pandemic [4, 61].Our survey was conducted late into the outbreak of the COVID-19 pandemic in China. The low infection rate (daily infections below 20) does not mean that the changes COVID-19 has had on people’s lives can be ignored. College students continue to experience the strain of the pandemic.

Moreover, this study found that PHTP mediated the effects of the perceived impact of COVID-19 on mental health. Specifically, it demonstrated a tendency whereby individuals who self-reported being more impacted by COVID-19 were more likely to hold PHTP, which might increase the risk of ill-being, but also slightly increase well-being. The PHTP demonstrated adaptability to well-being under the influence of the FTP and self-control model, which was consistent with the findings of previous studies [23]. The possible explanation is that PHTP may still be a protective factor for well-being, but this protection is inadequate in the pandemic, for the reason that PHTP also increases the risk of ill-being and negative psychological outcomes [31]. In particular, PHTP further reduces well-being and increases ill-being under the multiple mediating effects of self-control [23].

Furthermore, this results showed that only the mediating effects of FTP between perceived impact of COVID-19 and well-being rather than ill-being was significant. Interestingly, we found that the FTP had a significant positive impact on well-being, which did not show the depletion of enjoyment as previous studies did [23, 46]. A possible explanation is that focusing on the future may make people feel more hopeful and optimistic during the pandemic than ever before [47], which significantly increases their well-being.

Unlike the findings of previous studies whereby individuals with FTPs experienced less negative affects [33], having an FTP may not reduce negative emotion and anxiety directly. One possible reason involves the context of COVID-19 in that when individuals are generally under stress, they experience more negative emotions than before. Another possible explanation is that well-being and ill-being are two different constructs contrary to two ends on a continuum [47]. The enhancement of well-being does not mean the reduction of ill-being, which confirms the importance of considering both well-being and ill-being simultaneously. Furthermore, we established that the FTP can positively predict well-being directly, but the impact of the FTP on ill-being was fully mediated by self-control. In the face of the negative impacts of the COVID-19 pandemic, the findings of this study indicated that simply emphasizing the utility of emphasising only the FTP is limited. Therefore, the relationship between the FTP and self-control must be considered.

Additionally, the results of this study demonstrated that self-control had a direct mediating effect underlying the effects of perceived impact of COVID-19 on well-being and ill-being. Environmental changes may directly reduce self-control among individuals, thereby increasing the risk of ill-being and reducing the benefits of well-being. In our TP and self-control model, self-control was a crucial protective factor for well-being during the COVID-19 pandemic, and it functioned through a mediating mechanism, as demonstrated through our mediation model (well-being and ill-being), whose results differ from the findings of a previous study showing a moderating effect [22]. This result supported the motivational explanation of self-control [62]. Thus, we consider that self-control is an option based on value and various internal cues, such as emotions, demands, and beliefs and various external cues, such as motivations, social pressure, and the environment [32].

### Implications and Limitations

This study provides further insight and theoretical contributions. Firstly, it is a new attempt to individual’s mental health from time perspective in social psychology, which providing a new perspective for understanding the factors affecting well-being and ill-being in the pandemic. To be specific, PHTP and FTP can reflect human trade-offs in face of the uncertainty brought about by the pandemic, which will further affect mental health [10, 22]. Secondly, the results provided empirical evidence regarding the dual-factor model of mental health in which well-being and ill-being are two different structures, as opposed to two ends of one dimension[6] [63]. Thirdly, the relationship between the FTP or the PHTP and self-control has been proven and enriched in the context of pandemic by a multiple mediation model. This study provides a deeper understanding of the motivational interpretation rather than the ability-related interpretation of self-control, as it pertains to TPs. Specifically, the multiple mediating effects of the FTP and self-control have a significant impact on ill-being, thereby indicating that the association between the FTP and self-control has a high protective effect in the current COVID-19 pandemic.

In addition, this study provides some practical implications for public health management and clinical interventions. In terms of policy publicity, excessive emphasis on the negative effects of COVID-19 may reduce self-control among college students, thereby impairing their mental health. Therefore, emphasizing future changes brought about by active pandemic prevention and exhibiting confidence in the future may help individuals to enhance confidence and increasingly comply with pandemic prevention policies. Meanwhile, psychological interventions regarding time perspective can help reduce psychological distress and increase well-being during the pandemic. For example, emphasize long-term goals, distance yourself from current impulses, and commit to actions that increase self-control. These measures will help college students to reduce negative emotions and improve their well-being during the pandemic. These suggestions also provide reference for countries having a similar structure or measures defeating epidemic with China, so as to better cope with future changes in the epidemic.

This study has several limitations, and several research directions can be pursued in the future. Firstly, the cross-sectional design does not allow for conclusions regarding causation. Thus, additional lab-based experiments are required to manipulate participants’ TPs in laboratory environments, or a longitudinal study design may be necessary to examine the ongoing effects of TPs on mental health in the future. Secondly, the convenience sampling might limit the generalisation of our findings beyond those involving Chinese college students. In the future, samples from multiple countries and cities are needed to improve the representativeness of samples to verify the stability of research results. Thirdly, based on the influence of the COVID-19 pandemic on TPs, only the FTP and the PHTP were considered in the model. Other types of TPs could be included in future studies to clarify the change process and influence mechanism of different TPs during the epidemic. Fourthly, this study revealed the effect of TPs on mental health in COVID-19 by constructing a multiple mediation model, which provides empirical evidence for the influence mechanism of time perspective and well-being. However, it cannot be clarified the changing conditions of this influence mechanism in this study. Future studies could focus on exploring additional moderating variables, such as social support and personality factors, to better illustrate the influence of COVID-19 on mental health.

## Conclusion

In conclusion, this study elucidated how TPs changed and affected college students’ mental health when facing the uncertainty due to COVID-19. It enriches the theoretical framework of environmental adaptive function of TPs, and provides practical implications for psychological interventions during the COVID-19 pandemic. The mediating effects of FTP and self-control may relieve the psychological distress and enhance the happiness of college students during the pandemic in China. Maintaining an FTP and improving self-control could be a good medicine to boost individual well-being in the COVID-19 pandemic.

## Electronic supplementary material

Below is the link to the electronic supplementary material.

Supplementary Material 1

## Data Availability

The datasets used in this study are available from the corresponding author on reasonable request.

## List of abbreviations

TP: time perspective.
FTP: future time perspective.
PHTP: present-hedonistic time perspective.
SC: self-control.
PA: positive affect.
NA: negative affect.
