# Supplementary material for: Role of time perspectives and self-control on well-being and ill-being during the COVID-19 pandemic: A multiple mediation model
Source: BMC Psychol. 2022 Oct 27;10:238. doi: 10.1186/s40359-022-00933-2 (PMC9610352; doi:10.1186/s40359-022-00933-2)
Supplement: Supplementary file 1 — Supplementary Material 1 [file 40359_2022_933_MOESM1_ESM.docx]

**Role of time perspectives and self-control on well-being and ill-being during the COVID-19 pandemic:**

**A** **multiple mediation model**

**Supplemental Materials**

***Validation of Perceived*** ***impact of COVID-19(PI) Questionnaire***

**Method**

***Measurement***

The PI questionnaire includes 6 items on a 5-point Linkert scale (1 = *large positive impact*; 5 = *large negative impact*). The score ranged from 6 to 30, with higher score indicating higher perceived negative impact of COVID-19.

***Analytic approach***

All statistical analyses were conducted using IBM SPSS 24.0 and Mplus 8.0. In order to evaluate the construct validity of the independent variable Perceived impact of COVID-19 (PI) in this study, the researchers split the sample randomly half by half. An exploratory factor analysis (EFA) including half of the sample was conducted using Principle Component Analyses (PCAs) and Direct Oblimin rotation. A Confirmatory Factor Analysis (CFA) including the other half of the sample was performed by Maximun Likelihood (ML) estimates to confirm the fitness of the model derived from EFA. The goodness of model fit was evaluated by a number of statistics, i.e., chi-squared-degree of freedom ratio (χ2/df), root mean square error of approximation (RMSEA), comparative fit index (CFI), Tucker-Lewis index (TLI), and Standardized Root Mean Residual (SRMR) [1]. Acceptable goodness-of-fit model parameters were defined as RMSEA < .08, CFI > .90, TLI > .90, SRMR < .08 [2].

**Results**

***Results of EFA***

In the first data subset of 962 participants, The Kaiser–Meyer–Olkin (KMO) measure and Bartlett’s test of sphericity were employed to test the EFA. KMO= 0.78, indicating that the data were adequate for EFA [3]. Bartlett’s *χ*^2^ (15) = 1929.58, *p* < .0001, indicating that it was acceptable to proceed with the analysis [4]. Based on the criterion of Eigenvalues being greater than 1, two factors were exacted, accounting for 69.03% of the total variance, suggesting a two-dimension solution. The correlation between the two factors is 0.56 (*p* < .001). All items showed low cross loadings, and all loadings were greater than 0.40. Detailed factor loading and community for each item was shown in Table 1S.

Table 1S. Factor loadings of PI items

| **Items** | **Factor 1** | **Factor 2** | **Community** |
| --- | --- | --- | --- |
| 1 To what degree have your studies been impacted by COVID-19？ | 0.807 |  | 0.682 |
| 2 To what degree have your application for employment/internship been impacted by COVID-19？ | 0.800 |  | 0.705 |
| 3 To what degree have your examination been impacted by COVID-19？ | 0.883 |  | 0.819 |
| 4 To what degree have your love relationship been impacted by COVID-19？ |  | 0.765 | 0.623 |
| 5 To what degree have your friendship been impacted by COVID-19？ |  | 0.815 | 0.692 |
| 6 To what degree have your entertainment and leisure life time been impacted by COVID-19？ |  | 0.755 | 0.621 |
| **Explained variance (Total: 69.03%)** | 36.45% | 32.58% |  |

*Note*. PI: Perceived impact of COVID-19

***Results of CFA***

The remaining half sample of 962 participants was used to conduct CFA. The following series of models were evaluated: a one-factor model, a two-factor model derived from EFA, and a higher-order model. Fit statistics for the three models were shown in Table 2S.

Table 2S. Model fit indices for the measurement model

|  | *χ*^2^(*df*) | RMSEA | CFI | TLI | SRMR |
| --- | --- | --- | --- | --- | --- |
| Model 1 (one-factor) | 750.096 (9) | 0.207 | 0.799 | 0.665 | 0.094 |
| Model 2 (two-factor) | 71.548 (8) | 0.064 | 0.983 | 0.968 | 0.024 |
| Model 3 (Higher-order) | 71.584 (7) | 0.069 | 0.983 | 0.963 | 0.024 |

Based on the fit criteria mentioned above (CFI > 0.9, TLI > 0.9, SRMR < 0.08, RMSEA < 0.08), the one-factor model did not fit the data, and the two-factor model derived from EFA showed the best fitness. Although the high-order factor model also showed a good fitness, the specific indicators were not better than the two-factor model. Based on the aim of this study focusing on the theoretical model rather than validity, the two-factor model was selected for the following structural equation modelling.

***Reliability***

The reliability of the 2-factor model was tested using the sample size of 1924. This self-made questionnaire showed a good internal consistency reliability. As shown in Table 1S, the total Cronbach’s α = 0.80. The Factor 1 was named ‘PI academic’ (Cronbach’s α = 0.817), and the Factor 2 was named ‘PI life’ (Cronbach’s α = 0.718).

***Measurement model***

**Parcelled self-control CFA**

Three parcels were constructed to estimated self-control variable by the domain representative technique, in which items from each dimension were combined until no items remained [5]. We summed one parcel from each dimension into one indicator, a second parcel from each dimension into a second indicator and so on until no items remained [6, 7]. Parcels 1 includes items 5, 6, 10, 14, 15, 16 and 18; Parcels 2 includes items 3, 9, 11, 12, 13 and 19; Parcels 3 includes items 1, 2, 4, 7, 8 and 17, respectively. CFA for Parcelled self-control was conducted using randomly split data [7]. Model was just-identified, *χ*^2^(0) = 0.00, *p* = 0.00; RMSEA = .00; CFI = 1.0; TLI = 1.0 (See Figure 1S).


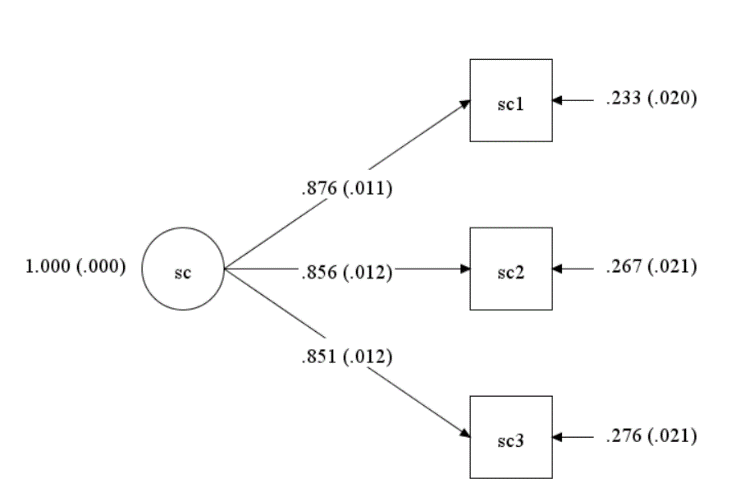


Figure 1S. Parcelled self-control Confirmatory Factor Analysis (*N* = 962)

**References**

1. Hu L, Bentler P. Fit indices in covariance structure modeling: Sensitivity to underparameterized model misspecification. Psychological Methods. 1998;3:424-453.

2. Wen Z, Hau K, Herbert WM. Structural equation model Testing:Cutoff criteria for goodness of fit indices and Chi-square test. Acta Psychologica Sinica. 2004;36(2):186-194.

3. Kaiser H, Rice J. Little Jiffy, Mark Iv. Educational and Psychological Measurement. 1974;34(1):111-117.

4. Bartlett MS. A note on the multiplying factors for various chi-square approximations. Journal of the Royal Statistical Society: Series B. Methodological. 1954;16:296–298.

5. Kishton JM, Widaman KF. Unidimensional Versus Domain Representative Parceling of Questionnaire Items: An Empirical Example. Educational and Psychological Measurement. 1994;54(3):757-765.

6. Kim J, Hong H, Lee J, Hyun M-H. Effects of time perspective and self-control on procrastination and Internet addiction. Journal of Behavioral Addictions. 2017;6(2):229-236.

7. Mcclanahan W, Van Der Linden S, Ruggeri K. Decision-making style mediates the relationship between trait self-control and self-reported criminal behavior. Personality and Individual Differences. 2019;151.
